# Integrated primary health care services in two protracted refugee camp settings at the Thai–Myanmar border 2000–2018: trends on mortality and incidence of infectious diseases

**DOI:** 10.1017/S1463423622000044

**Published:** 2022-03-22

**Authors:** Oliver Mohr, Marie T. Benner, Ammarat Sansoenboon, Wiphan Kaloy, Rose McGready, Verena I. Carrara

**Affiliations:** 1 Independent Consultant, Bad Krozingen, Heidsteinweg, Germany; 2 Independent Consultant, Streithausen, Am Baumort, Germany; 3 Malteser International, Ban Kad, Mae Sariang, Thailand; 4 Shoklo Malaria Research Unit, Mahidol-Oxford Tropical Medicine Research Unit, Faculty of Tropical Medicine, Mahidol University, Mae Sot, Thailand; 5 Centre for Tropical Medicine, Nuffield Department of Medicine, University of Oxford, Oxford, UK; 6 Global Health Institute, Department of Medicine, University of Geneva, Geneva, Switzerland

**Keywords:** infectious diseases, mortality, primary health care, refugees, south east Asia, Thai-Myanmar border

## Abstract

**Aim:**

This study aimed to assess the health outcome of four epidemic-prone infectious diseases, in the context of a Primary Health Care project implemented in a protracted refugee setting along the Thai–Myanmar border.

**Background:**

Refugees settled at the Thai–Myanmar border are fully dependent on support for health services, shelter, food, education, water, and sanitation. The Non-Governmental Organization Malteser International developed an integrated Primary Health Care program in close cooperation with trained camp residents over 25 years in the two settlements under its supervision. The project has been funded by the European Commission Civil Protection and Humanitarian Aid Operations (DG ECHO).

**Methods:**

This was a retrospective primary health care project evaluation. All-cause mortality; morbidity trends in malaria, lower respiratory tract infections (LRTIs), watery diarrhea, and dysentery; and health service utilization covering a time span of 18 years were assessed. Programmatic changes in the Primary Health Care (PHC) project and events with a potential effect on health of the target population were examined.

**Findings:**

Despite the continuous drain of trained health care workers, the volatile influx of refugees, and the isolated location of the two camps, the initial basic curative health care developed into an integrated and comprehensive PHC project including a SPHERE-compliant water, sanitation, and hygiene program. Malaria, LRTIs, watery diarrhea, and dysentery morbidity dropped twelve, three, two, and fivefold, respectively, over the 18-year period evaluated while the health services utilization dropped from 7.1 to 2.9 consultations per refugee/year. The international community may face situations where integration of refugees into the health services of the host country is not possible. In such a context, integrated and evidence-based PHC adequately funded and implemented by one health agency is an effective and relevant approach to reduce the infectious diseases burden under the constraints of semipermanent living conditions.

## Background

**Figure 1.**
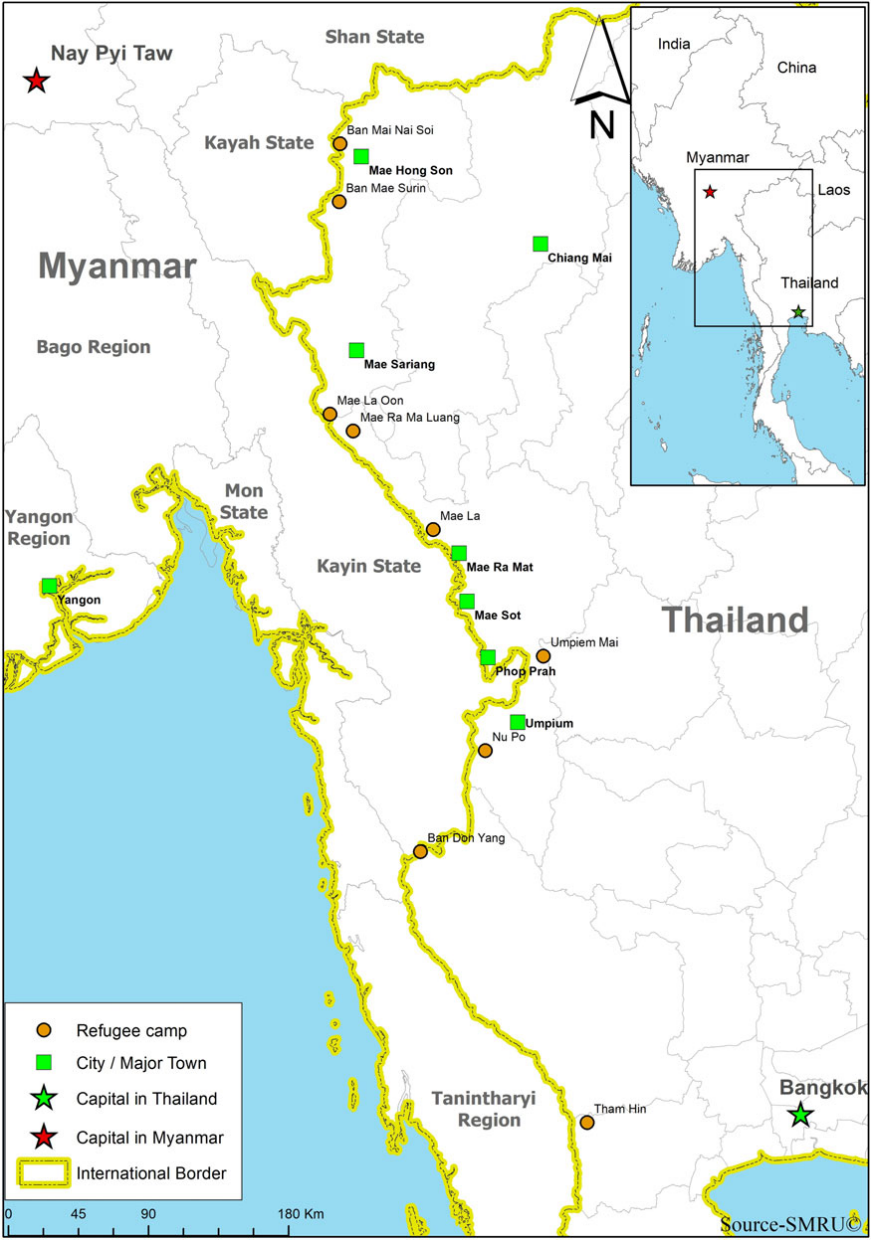
Map Thai–Myanmar border. Source: Shoklo Malaria Research Unit, Mae Sot, Thailand

For more than 30 years, refugees from Myanmar have been fleeing to Thailand to seek refuge from human rights abuses by the authoritarian regime in Myanmar and from fights between armed opposition groups and the Myanmar military. On the Thai side of the border, camps for displaced people residing primarily in Kayin State (southern and southeastern Myanmar) and predominantly of the Karen ethnic group were established in 1984. At the end of 2018, approximately 87 000 refugees from Myanmar were housed in nine official camps along the border, from Mae Hong Son Province in the north to Ratchaburi Province, southwest of the Thai capital of Bangkok (Figure 1). These refugees are fully dependent on support for health services, shelter, food, education, water, and sanitation. Several local and international Non-Government Organizations (NGOs) supply services in close cooperation with trained camp residents in order to ensure access to basic needs (Benner *et al.*, 2008).

Malteser International (MI) offers health services, water, and sanitation in the refugee camps of Mae La Oon (MLO) and Mae Ra Ma Luang (MRML) in the southern part of Mae Hong Son Province. The two camps are located in an isolated tropical forest at the Yuam river, 80 km from Mae Sariang, the nearest hospital with operative capability, and 4 km from the Myanmar border. In 2018, MLO and MRML camps harbored circa 10 000 refugees each, which made them among the most populous camps after Mae La and its 36 000 refugees (The Border Consortium, 2018). Both camps are difficult to access, especially during the rainy season, and prone to natural disasters and infectious diseases outbreaks. MI health projects aim to reduce the risk of epidemics and strengthen the capacity and capability of the camp community for self-reliance and sustainability.

This study describes the development and evolution of the health care support in those two protracted refugee camps over a 18-year period, from basic curative care to a comprehensive Primary Health Care (PHC) program; the challenges in maintaining such a program; the trends in all-cause mortality and in four infectious diseases targeted by the various components of the PHC during that time period, namely lower respiratory tract infections (LRTIs), malaria, watery diarrhea, and dysentery. The four diseases were selected since they are epidemic-prone, responsible for most hospitalizations, and have standardized clinical guidelines.

## Methods

### Clinic setting

The PHC program at MLO and MRML camps relies on local staff themselves, camp residents themselves, and able to communicate in local languages (Sgaw and Poe Karen, and Burmese) as well as in English. The medical staff (herein referred to as medics) who run the outpatient departments (OPDs) and inpatient departments (IPDs) need to attend a 6-month theoretical curriculum-based training followed by 5 months practical, supervised internship in the clinics. The medics curriculum follows the Burmese Border Guidelines (Shoklo Malaria Research Unit, 2016), the midwives need to attend 6 months theory and receive 6 months supervised practice, laboratory technicians and nurses receive a 6-month training (3 months theory and 3 months practice), and community health workers (CHWs) are trained for 10 d. After completion of all training modules, health staff must pass a final examination. Supervision for each work category is conducted by Thai-registered professionals (nurse trainer, midwive, lab technician, medical doctor). To retain staff and reward services, the health staff receive financial compensation based on experience and responsibility.

In both camps, each OPD is equipped with three medics and three nurses in the daytime (8am – 5pm); at night, medics in charge of IPD cover emergency cases. IPDs maintain 24 h coverage and are staffed with 2–3 medics per shift (day, evening, and night shift). A medically qualified doctor supports the staff in the camps, but is not permitted to enter the camps on weekends.

Before 2000, the PHC project in the two camps focused on curative health services. Over the years, the project became comprehensive: preventive services including Information, Education and Communication (IEC) strategies to promote behavior change were applied; a Mother and Child and Adolescent Health (MACH) component was added; data collection and analysis were improved; various treatment guidelines were established; and collaboration with other health NGOs at the Thai–Burmese border was intensified. Water, sanitation, and hygiene (WASH) including vector control and central elements limit the risk of communicable diseases in populations in any emergency or protracted crisis, became an important pillar of the project.

### Data collection

#### Health services and training activities

Service provision and training activities in MLO and MRML camps, as well as events with a potential effect on health, were retrospectively reviewed from MI annual reports spanning 18 years. Relevant health interventions were defined as programmatic changes of the WASH component, vector control, and training of health care workers. Type and duration of WASH and of vector control measures were extracted from the annual reports’ narrative and transposed on a timeline matrix that included the occurrence of infectious diseases outbreaks and external events potentially influencing health outcomes (Table 1).

**Table 1.** Timeline matrix

Year	Health interventions	WASH interventions and vector control	Infectious disease outbreaks	Events
2000	Vaccines for EPI provided by Thai MoPH (before purchased by MI)	WASH: Installation of water flush latrines; start to replace the common bamboo pipes by PVC pipe water systemVector control: residual indoor spraying; provision of impregnated bed nets by TBC		
2001		WASH: Provision of soap 70 g/pers./month; continued development of PVC pipe water system		
2002		WASH: Installation of ceramic filters at end-use point	Measles outbreak	Flood in MLO camp: 26 people died, major damage to camp infrastructure (houses, schools, nurseries, offices, and OPD clinics)
2003	MI takes over the PHC program in MRML camp from AMI	WASH: >90% of HH covered with flush latrines; soap provision increased to 125 g/pers./month		
2004		WASH: external water quality control 4 samples/year		MLO camp relocated to a site 80 km away to a national preserved park area prone to landslides
2005	ARV provision started by Thai MoPHIntensified training of CHW, on pharmacy management, case definitions, and rational use of antibiotics	WASH: construction of 500 000 L water tank (concrete pond) and reinstallation of water supply PVC pipes after relocation of 400 HH (*only* MLO camp)		Start of UNHCR resettlement programVanished antibiotics in the MRML hospital pharmacy, replacement of hospital, and pharmacy managementTensions between MI and MRML camp population Security issues
2006				First staff members resettled
2007	Intensified training and supervision of CHW on early diagnosis and adequate treatment for malaria;Systematic malaria screening for all new arrivals in camps	WASH: Start of water chlorination as prevention measurement due to cholera outbreak in Mae Sot District	Cholera outbreak in Mae Sot District, but no cases in MLO and MRML campUnusually high number of malaria-related deaths (*n* = 6)	Ongoing resettlement of health staff
2008		WASH: poor maintenance of ceramic filters led to an increased risk of contamination; filters were abolished; 30 rainwater collection facilities (2500 L fiber glass water tanks) installedVector control: MI takes over bed net impregnation (with deltamethrin/permethrin) and distribution from TBC	Measles outbreak (*only* MLO camp)	Ongoing resettlement of health staff
2009	Start of EuropeAid project supporting training activities with the title “Health Care Project for uprooted people in Sop Moi District” up to 2012. This project aims at ensuring the provision of quality of health care for refugees and at strengthening the Thai health care system by linking both health care providers together	WASH: 99% flush latrine HH coverage accomplished		Ongoing resettlement of health staffFighting and unrest in Myanmar
2010		Vector control: Avian influenza and A/H1N1 preparedness program established; ongoing indoor spraying	Influenza A/H1N1 outbreak	Ongoing resettlement of health staffIDP camp (4000 pop.) across the border uses the health services in the camps Unrest in Myanmar
2011	ARV cost coverage by Thai NPhA stopped; WHO/MoPH surveillance project starts; EuropeAid starts funding WASH additional to ECHO funding	WASH: construction of 7000 L cement tanks (*only* MRML camp and Thai villages)Vector control: mobile bed nets for mobile camp pop.; closer co-operation with provincial PH office on outbreak control		Ongoing resettlement of health staffContinued unrest in MyanmarFlooding in both camps destroys health infrastructure
2012	GFATM starts funding malaria through SMRU (2016): Provision of support in human resources, lab consumables, lab equipment and maintenance, malaria campaigns, and personal protection equipment	WASH: external water quality control with 8 samples/yearVector control: Provision of hammock nets and repellents		Ongoing resettlement of health staffMedical doctor and health coordinator position at field office vacant for few months
2013	Start of EuropeAid project for surrounding Thai villages & fund WASH/Health until 2017JE and MMR vaccination startedStart of “Camp Health Advisory Group”: Encourage community in taking more responsibility for analyzing health problems in the camps and getting engaged in planning of preventive and educational activities	WASH: 100% HH coverage with 20 L buckets (incl. Thai villages); 10 chlorine dosing units; outsourcing of waste management through camp population; setting up local soap production; provision of 250 mg soap for the most vulnerable persons, handwashing points in 8 schools	Measles outbreak (*only* MRML camp); delay in measles vaccines supply for new arrivals	Ongoing resettlement of health staffFrequent border crossing of camp residents
2014				Ongoing resettlement of health staffClosure of 2 hospitals (out of 4)
2015			Measles outbreak (*only* MRML camp)Hepatitis A outbreak	Ongoing resettlement of health staff
2016		WASH: Prevention campaign on Hepatitis A including camp restaurants, closure of schools; closure of camps to visitors	Ongoing Hepatitis A outbreak	Ongoing resettlement of health staff
2017			Influenza A/H1N1 outbreak	Ongoing resettlement of health staff
2018		WASH: external water quality control with 32 samples/year		Landslide in MLO camp

AMI: aide médicale internationale; ARV: antiretroviral therapy; CHW: community health worker; ECHO, European Civil Protection and Humanitarian Aid Operations; EPI: extended program of immunization; GFATM: the global fund to fight; HH: household; JE: Japanese encephalitis; L: litre; MLO: Mae La Oon; MMR: measles, mumps, rubella; MoPH: ministry of public health; MRML, Mae Ra Ma Luang; NPhA: national pharmaceutical association; PH: public health; PVC: polyvinyl chloride; SMRU: Shoklo malaria research unit; TBC: Thai Burmese consortium; WASH: water, sanitation, and hygiene; WHO:World Health Organization.

Interventions and events occurred within the same time period in both camps, unless stated otherwise.

#### Population

Demographic data were obtained from the MI electronic database. The pooled annual mean of MLO and MRML camps population was used as the denominator in the analysis.

#### Mortality, morbidity, and program indicators

All-cause mortality and disease-specific morbidity data from both camps were extracted from the MI electronic database, which contains over 100 programmation indicators as well as all-cause mortality and new episodes of disease which are reported monthly, and aggregated by children under 12 months, children under 5 years, women aged 15–49 years, and total population.

Case definition for the four epidemic-prone infectious diseases selected for this analysis has remained constant over the years, and the local medical team was routinely trained to diagnose them correctly (Table 2).

**Table 2.** Case definitions of the four epidemic-prone infectious diseases selected

Malaria	Positive laboratory test for malaria parasites: Identified asexual form of *Plasmodium spp.* from blood smear (thick film or thin film) or screening test positive for *Plasmodium spp.* (Guidelines for Disease Surveillance 2012)
Lower respiratory tract infection	Clinical diagnosis: Fever and cough and abnormal chest sounds (Shoklo Malaria Research Unit, 2016)(Chest X-ray can confirm a pneumonia if diagnosis is not clear, however only available in secondary health care outside the camps)
Watery diarrhea	Clinical diagnosis: Three or more loose stool or one watery stool in the past 24 h with or without dehydration (Guidelines for Disease Surveillance 2012)
Dysentery	Clinical diagnosis: Acute diarrhea with visible mucous-bloody stool or presenting with WBC and RBC in stool under microscopic examination (Guidelines for Disease Surveillance 2012)

The number of new outpatient consultations and three WASH indicators was collected: the average daily quantity of potable water per refugee; the distance to the next water point; and the number of persons per latrine.

### Statistical analysis

As this study describes changes spanning 18 years and activity implementation occurred at the same time in both camps, both camps’ mortality and morbidity data were aggregated and reported by year rather than monthly. Crude mortality rate (CMR) was reported as the total number of deaths occurring in both camps divided by the combined annual mean camp population and multiplied by 1000 in a given year.

Incidence rates were measured as the number of new disease-specific cases occurring in both camps divided by the combined annual mean camp population and multiplied by 1000 in a given year.

The health services utilization was estimated by calculating the average annual (all-cause) consultation number per refugee and per year, both camps combined.

The three WASH indicators were compared to those set by SPHERE (Sphere, 2018), which has identified a set of minimum standards applied in humanitarian response (Table 3).

**Table 3.** The three WASH indicators selected and their objectives

WASH indicators	Objectives set by SPHERE (Sphere, 2018)
Average quantity of potable water	≥ 15 L/d
Distance to the next water point	< 500 m
Camp latrines	≤ 20 persons per latrine

The 95% confidence intervals of proportions were calculated using Wilson’s method (Brown *et al*., 2001).

### Findings

The main programmatic changes and relevant external factors occurring between January 2000 and December 2018 are displayed on a timeline matrix (Table 1).

### Population influx and brain drain of health staff

**Figure 2.**
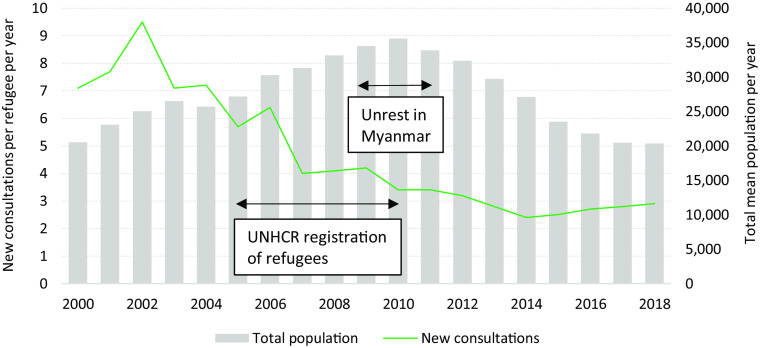
Annual mean population variations and utilization of health services in Mae La Oon and Mae Ra Ma Luang camps combined, 2000–2018.

Following the reintroduction of a resettlement program in 2005; the pooled MLO and MRML camps annual mean population increased by 11.4% in 2006, with 3110 additional refugees registered; in 2009, and for the following 2 years, political unrest in Myanmar led to a renewed population influx which reached its peak in 2010 with an annual mean population of 35 569 refugees (Figure 2). That year, United Nations High Commissioner for Refugees (UNHCR) closed the registration to new arrivals and the population rapidly declined as a combination of reduced influx and departures for resettlement to finally stabilize in 2017 at circa 20 000 a population similar to that documented in 2000.

The resettlement program led to a substantial drain of health staff: by 2007, the totality of the two camps health staff (*n* = 264) had applied for resettlement: in 2013 alone, MI lost 67 camp staff to resettlement and 20 more resigned due to other reasons (mostly related to repatriation). By 2017, 40% of MI camp-based workforce had left. In order to maintain the SPHERE minimum requirement of 1–2 CHW per 1000 people (Sphere, 2018), recruitment of new health workers and intensive training programs were set up. In 2012 and 2013, 97 newly hired staff were trained (36.7% of MI camp based workforce), and to better manage health care functionality, and in 2014, IPD services were merged from two IPD’s in each camp to one central IPD in each camp to assure quality health care services due to the loss of experienced staff and a slowly declining population. Taking into account the wide-spread camp area and the refugee’s need to be able to avail basic health care on a daily basis, two OPD’s per camp remained and continued to deliver services so that both SPHERE indicators “one healthcare facility by 10 000 people” and “80% of the target population can access primary healthcare within 1 h walk from dwellings” were still met (Sphere, 2018).

### Utilization of health services and WASH activities

The use of health services dropped from 7.1 to 2.9 consultations (new visits) per refugee/year between 2000 and 2018 (Figure 2), despite population fluctuations, the centralization of health care into one hospital per camp, and the occurrence of floods and landslides that destroyed health infrastructures (Table 1). Unemployment and boredom in the two camps and expectations upon health workers to provide medication might have contributed to the higher number of consultations than necessary observed in the earlier years, which also resulted in inappropriate prescription of various medications, especially painkillers and uncontrolled use of antibiotics. This led to a change in hospital management, an increased effort in pharmacy management, and, in 2005, further training on case definitions and rational use of antibiotics.

The WASH program in both camps underwent a well-documented development during the 18-year period, unhindered by the ongoing changes of CHWs. In 2000, the targets of a provision of ≥ 15 L potable water per refugee/day and a walking distance of less than 500 m from a water source were both already reached. From 2000 onwards, the program intensified access to water flush latrines and improved the piping system so that the SPHERE indicator of ≤ 20 persons per latrine was reached in 2003 and maintained with the near totality of households having access to a water flush latrine by 2009 (Table 1). In 2013, households were equipped with 20 L buckets while local capacity for soap production was developed.

An integral part of the program was health promotion and education, but also, when needed, the implementation of more drastic measures: in 2010, both camps went into complete lockdown in response to an outbreak of influenza A/H1N1 (Table 1).

### Programmatic interventions and changes in mortality and morbidity

**Figure 3.**
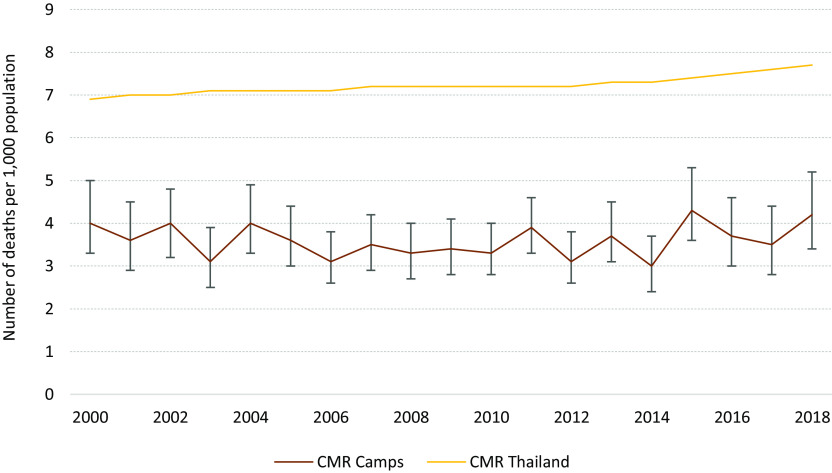
Trends in all-cause mortality rate (with 95% CI) for the all population (CMR) in Mae La Oon and Mae Ra Ma Luang camps combined, compared to that in Thailand, 2000–2018.

The CMR remained unchanged, varying between 4.0/1000 (95% CI 3.3–5.0) refugees in 2000 and 4.2/1000 (95% CI 3.4–5.2) refugees in 2018 (Figure 3).

### Diarrheal diseases

**Figure 4.**
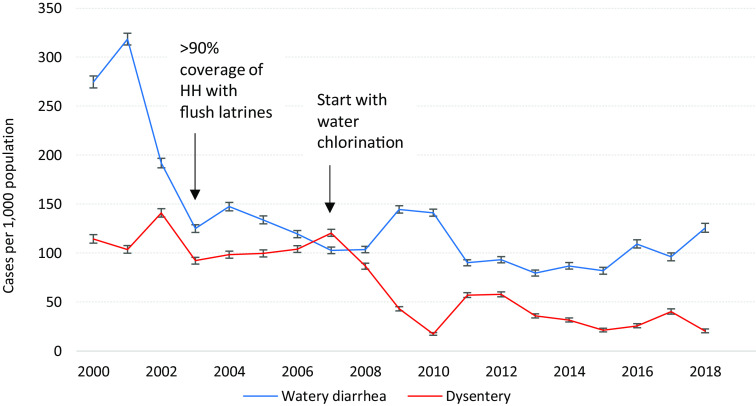
Trends (with 95% CI) in watery diarrhea and dysentery in Mae La Oon and Mae Ra Ma Luang camps combined, 2000–2018.

Watery and bloody diarrhea morbidity decreased substantially over the years; by 2003, 90% of camp households had access to a flush latrine and in the same time-period incidence of watery diarrhea declined by half, from 274.8/1000 (95% CI 268.7–280.9) in 2000 to 125.8/1000 (95% CI 121.3–130.4), *P* < 0.001. Since changes in watery diarrhea incidence have been minimal despite intensification of WASH activities. In contrast, incidence of dysentery remained unchanged until 2007; that year, water chlorination and intensification of control measures were introduced following the announcement of a cholera outbreak among Myanmar migrants in the neighboring province. This resulted not only in the absence of cholera transmission within MLO and MRML camps but also coincided with the sharp decline in dysentery cases which dropped more than 80% from 120.6/1000 (95% CI 117.1–124.3) in 2007 to 20.6/1000 (95% CI 18.7–22.6) in 2018 (Figure 4).

### Lower respiratory tract infections

**Figure 5.**
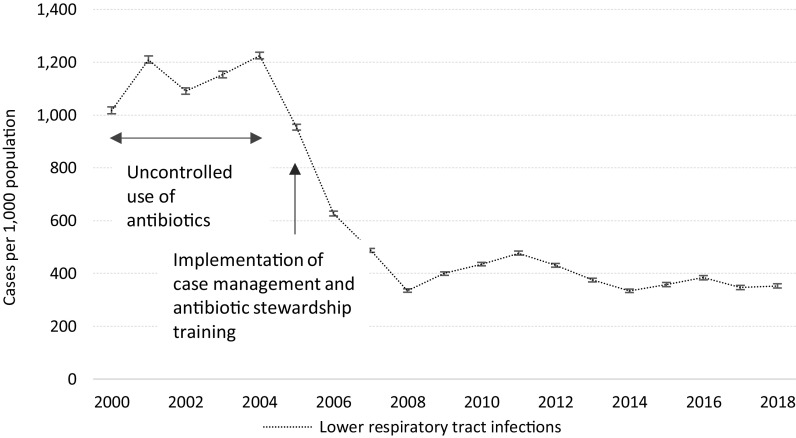
Trends (with 95% CI) in lower respiratory tract infections in Mae La Oon and Mae Ra Ma Luang camps combined, 2000–2018.

The incidence of LRTIs remained above one LRTI per camp resident per year between 2000 and 2004, followed by a sharp drop from 1225.5/1000 in 2004 to 335.4/1000 (95% CI 330.4–340.5) in 2008, coinciding with the introduction of trainings on case definitions and rational use of antibiotics (Figure 5). The confirmed uncomplicated 864 cases of influenza A/H1N1 seen during the September/October 2017 outbreak were counted separately from the number of LRTI. No casualty due to influenza A/H1N1 was recorded.

### Malaria

The distribution of impregnated bednets was uninterrupted, and indoor residual spraying campaigns continued throughout the 18-year period. The introduction, in the mid-nineties, of the combination of mefloquine and artesunate as first-line treatment for uncomplicated *Plasmodium falciparum* malaria in the two camps, had the largest impact on the global malaria burden reduction: from an all-species malaria incidence of 612.4/1,000 (95% CI 601.0–623.6) in 1996 (personal communication) to 108.4/1,000 (95% CI 104.2–112.7) in 2000.

**Figure 6.**
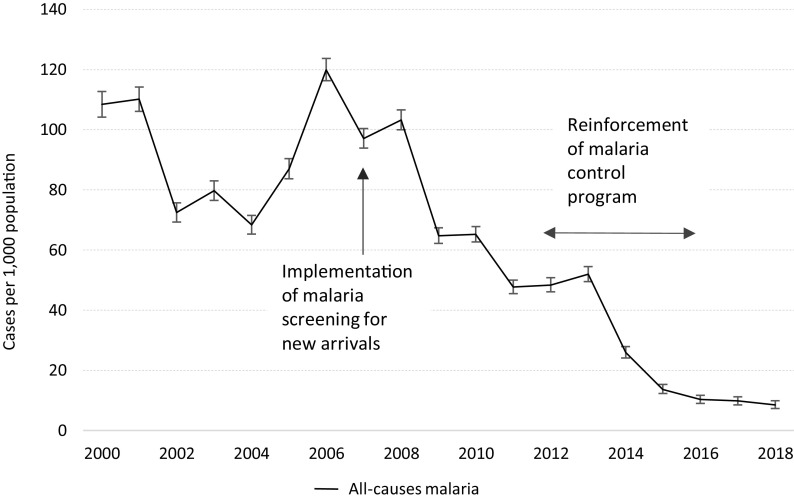
Trends (with 95% CI) in all-causes malaria in Mae La Oon and Mae Ra Ma Luang camps combined, 2000–2018.

In 2007, an unusually high number of malaria-related deaths (*n* = 6) compared to previous years (no malaria-related death in 2006 and one death in 2005) led to a detailed case investigation that showed that more than one- third of patients with severe malaria were not residing in the camps. Consequently, all new arrivals were systematically screened for malaria to reduce the risk of transmission within the camps population and medical staff and laboratory technicians received intensified training on malaria early diagnosis and adequate treatment and were closely supervised (Table 1). That, and secured funding dedicated to malaria, in the context of the malaria elimination in the Greater Mekong Region, led to a further reduction of both malaria species with all-cause malaria incidence declining to 8.5/1000 (95% CI 7.3–9.9) in 2018 (Figure 6 and Table 4).

**Table 4.** Changes in incidence of *Plasmodium falciparum* (PF) and *Plasmodium vivax* (PV) malaria in Mae La Oon and Mae Ra Ma Luang camps combined, 2000–2012^*^

	2000	2002	2004	2006	2008	2010	2012
Incidence PF malaria (per 1000 combined total population)	49.9	39.3	40.8	60.1	44.0	37.7	26.9
Incidence PV malaria (per 1000 total combined population)	36.5	27.8	26.4	58.3	65.4	28.8	21.1

* Data on PF and PV malaria by species were not available after 2012.

## Discussion

This overview spanning 18 years of an integrated PHC project, run by the same health agency, in two protracted refugee camps at the Thai-Myanmar border provides an insight of the successes and challenges encountered. The renewed political instability in Myanmar and the implementation of a resettlement program by the UNHCR had some major impacts on the camp’s population movements and the continuity of the health care program. As qualified, experienced, medics, and nurses were departing, there was a lack of mentorship for the new workforce. Despite the constant loss of health workers, the influx of new arrivals, the remote location of the camps, an important decline in the burden of the four most common and epidemic-prone infectious diseases was observed. The overall CMR was twice lower than that reported in Myanmar during the same period (9.1 in 2000 and 8.2/1000 in 2018) or in Thailand (12.2 in 2000 and 3.8/1000 in 2018) (The World Bank, 2021), and likely a reflection of the younger median age of the refugee population as observed in other protracted closed refugee settings (Spiegel *et al.*, 2001).

When humanitarian assistance and provision of health care for the refugees at the Thai–Myanmar border started in the eighties, it seemed unlikely that the refugee camps would still exist 40 years later. Emergency responses were first deployed with an emphasis on temporary solutions and a quick return of refugees to their homes; as the situation slowly settled, a more comprehensive program was installed although in the early years of the PHC project implementation, focus was in controlling infectious disease burden since communicable diseases such as diarrheal diseases, lower respiratory tract infections, and malaria account for the majority of morbidity in populations affected by complex emergencies (Black *et al.*, 2003; Connolly *et al.*, 2004). The focus on infectious diseases included the development and regular updates of concise clinical guidelines and reiteration of staff training to maintain the best quality of care possible despite the continuous loss of health staff and the lack of consistent mentorship. The development of a comprehensive WASH program for which the SPHERE indicators on water supply, sanitation (Sphere, 2018) were regularly monitored and met, contributed significanly to the improved health outcomes, in particular the reduction in diarrheal diseases.

Malaria control along the Thai–Myanmar border has always been a challenge due to the resistance of *Plasmodium falciparum* to most antimalarial drugs (Chareonviriyaphap *et al.*, 2000). In the mid-nineties, a combination of mefloquine and artesunate as a first-line treatment, combined with indoor-residual spraying, was introduced first in refugee camps of Tak Province that led to a sharp drop in *P. falciparum* malaria incidence (Nosten *et al.*, 2000). The dramatic decline in malaria burden observed in the camps paralleled with the decreasing malaria incidence in the migrant population on the Thai–Myanmar border (Carrara *et al.*, 2013), as did the increasing contribution of *P. vivax* to overall malaria morbidity (Baird, 2011; Douglas *et al.*, 2011; Imwong *et al.*, 2012). The decline of malaria *P. falciparum* incidence and the increasing contribution of *P. vivax* to overall morbidity was as well seen in Thailand as a whole; in 2018, only 447 cases of *P. falciparum* as opposed to 3575 cases of *P. vivax* were documented (WHO, 2019). As a Sub-Sub-Recipient of the “Global Fund to fight AIDS, Tuberculosis and Malaria” (GFATM), within the “Partnership of containment of Artemisin Resistance and Moving towards the elimination of Plasmodium Falciparum in Thailand”, MI benefited between 2012 and 2016 from additional financial support to reinforce its malaria control program and develop malaria-related health campaigns and production of IEC materials, and support in human resources, lab consumables, lab equipment including maintenance, impregnated bednets, hammock nets, and repellents.

Implementing such a PHC project raises inevitably the question of sustainability as it is not only depending on external funding but is also a parallel health system to the host country (Rowley *et al*., 2006). The integration of affected refugees into national health systems by addressing the humanitarian development nexus is seen as a useful approach (Spiegel, 2017) – if such an integration is not feasible, then a chronic situation of unpredictable duration develops and maintaining a certain harmony with the local host population can be facilitated by allowing them access to that parallel system; this has been the case in this context as the host Thai population estimated at 4000–5000 persons, mostly from the same ethnic background as that of the refugees population and residing in remote, rural areas far from available Thai services, benefited from camps health services, and refugees were permitted to use secondary and tertiary health care in Thai health facilities. While the health of the host population in protracted refugee settings is often neglected (Orach and de Brouwere, 2004), this was not so in this project and it may have increased the acceptance of the refugees by the local population. Nevertheless, sustainability remains an ongoing issue for refugee camps globally (Abbas *et al.*, 2018).

In addition, the health agency implementing a PHC project in such an unstable, post-conflict setting requires an extreme flexibility and adaptation to rapid population changes, as well as access to resources to provide continuous training programs to replace the continuous loss of staff and to secure the provision of adequate PHC.

Today, the curative services include not only in- and OPDs, but also services dedicated to patients with chronic health conditions, a small surgery facility including eye surgery and dental care. A referral mechanism to secondary and tertiary Thai health care is in place. The PHC system includes a MACH component, complementing the infectious diseases control program and the WASH program. Preventive services promote behavior change communication and awareness on all these different health aspects.

This evaluation is not without limitations. This study is observational, based on a retrospective analysis of changes in selected indicators as the PHC program evolves; no statistical comparison or measures of association were possible to ascertain the causality between the reduction of disease burden and the development of the PHC program; however, the selected diseases were those most likely influenced by the local implementation of control measures rather than by population or environmental changes; their case definition as well as their laboratory confirmation remained unchanged; and quality of malaria diagnosis was regularly assessed to maintain a specificity and sensitivity above 90%.

The denominator used to evaluate the changes in the incidence of disease burden was based on the pooled annual mean camps population only; as the host population and neighboring migrant communities from Myanmar also had free access to health care, the estimated incidence might have been biased. However, influx of new cases from camps outsiders was fairly constant and would not have greatly influenced the annual trends. Disease-specific data were not collected by gender and trends according to age categories could not be compared. Finally, as with any collected data, the assumption was that the information collected had been validated and therefore deemed correct.

## Conclusion

In conclusion, with the Sustainable Development Goals and the goal of universal health coverage in mind, an integrated and evidence-based PHC, if adequately funded and implemented by one health agency, likely contribute to reduce the infectious diseases burden in a protracted refugee camp setting where integration into the health services of the host country is not an option.
